# Real-life clinical benefit of oral metronomic cyclophosphamide administration in elderly and heavily pretreated epithelial ovarian cancer patients

**DOI:** 10.1007/s00404-024-07670-4

**Published:** 2024-08-02

**Authors:** Daniela Attianese, Roberta Massobrio, Margherita Giorgi, Michela Villa, Luca Fuso, Enrico Badellino, Marco Bellero, Annamaria Ferrero

**Affiliations:** 1 Academic Division of Gynecology and Obstetrics, University of Torino, Mauriziano Umberto I Hospital, Torino, Italy; 2 Department of Surgical Sciences, University of Torino, Torino, Italy; 3 Division of Gynecology and Obstetrics, Cardinal Massaia Hospital, Asti, Italy; 4 Pharmacology Division of Mauriziano Umberto I Hospital, Torino, Italy

**Keywords:** Oral metronomic cyclophosphamide, Elderly, Ovarian cancer, Clinical benefit

## Abstract

**Purpose:**

Oral metronomic cyclophosphamide (OMC) implicates the daily administration of low doses of chemotherapy. Its antitumor activity combined with an oral administration route and a good toxicity profile makes OMC an attractive option for heavily pretreated patients. We retrospectively evaluated OMC’s clinical benefit and objective response in recurrent ovarian cancer patients.

**Methods:**

This is a retrospective observational study involving patients treated with OMC (50 mg daily) from 2017 to 2022 at the Academic Division Gynaecology, Mauriziano Hospital, Torino, Italy. Clinical benefit assessment included CA125 response, radiological response, and reported symptomatic improvement. Toxicities were reported using Common Terminology Criteria for Adverse Events version 5.0.

**Results:**

Thirty-eight patients (average age 72, range 49–88) were included. 90% had FIGO stage III/IV at diagnosis and 64% underwent ≥ 3 previous lines of chemotherapy. Before OMC, 79% had ECOG 1 or 2. 8.6% of patients had a partial response (PR), and 40% a stable disease (SD). Median duration of response was 7.4 months. After 3 months on OMC, 51% experienced symptom improvement, and 53.3% experienced Ca125 reduction or stabilization. 66.7% of patients older than 75 responded to treatment; in 40% of cases, responses lasted ≥ 6 months (*p* = 0.08). No G3-4 hematological toxicities occurred. Nausea and fatigue G1–G2 were reported in 5 (13%) and 13 (34%) cases, respectively.

**Conclusion:**

OMC is a feasible therapeutic option for recurrent ovarian cancer, providing satisfying clinical responses with a good toxicity profile, even in elderly and heavily pretreated patients with a suboptimal performance status.

## What does this study add to the clinical work?

**Table:** 

Oral metronomic cyclophosphamide (OMC) is a feasible therapy for elderly and heavily pretreated ovarian cancer patients, due to its clinical benefit and good toxicity profile. Clinical benefit and quality of life assessment should be integrated in clinical practice.	

## Introduction

Debulking surgery and platinum-based chemotherapy are the cornerstones of ovarian cancer (OC) treatment. Complete surgical resection is the main independent prognostic factor, and platinum-based chemotherapy followed by maintenance treatment produces high response rates reaching 80%. However, in 75% of cases, disease recurrence occurs in the 2 years following primary treatment [1].

Treatment algorithms for recurrent ovarian cancer evolved in the past years, passing from being driven by the platinum-free interval (PFI) to be tailored on tumor biology, patient’s symptoms, persistent toxicities, response to previous treatments, PFI, and the potential response to platinum therapy. This complex evaluation is resumed by the concept of “platinum eligibility” (PE) [2]. Platinum non-eligible patients usually undergo single-agent therapies, such as weekly paclitaxel, pegylated liposomal doxorubicin, gemcitabine, and topotecan, with response rates around 10–15%, PFS of 3–4 months, and OS of approximately 12 months [3].

In addition to chemotherapy, bevacizumab and Poly(ADP-ribose) polymerase (PARP) inhibitors play a role in maintenance treatment in first line and recurrent settings. Immune checkpoint inhibitors are being tested in various contexts [4].

Despite the treatment landscape for recurrent ovarian cancer is quickly expanding, responses beyond the second line of therapy are progressively low. In addition, treatments after the fourth line seem not to be beneficial and predictive factors of response are not available [5, 6]. Therefore, in advanced lines, an adequate therapeutic strategy is required, and the advantages and disadvantages of chemotherapy should be balanced with previous toxicities and quality of life [7].

In this context, metronomic chemotherapy becomes a relevant treatment option, since it is well tolerated with an oral administration route. The term metronomic refers to the regular administration of therapeutic drugs, at doses that are well below the maximum tolerated dose [8]. Metronomic chemotherapy was initially considered an anti-angiogenic treatment strategy, but in the last decade, further preclinical studies have revealed other possible mechanisms, including the enhancement of the antitumor immune response and direct action on chemotherapy-resistant cancer cells [9–11].

Oral administration of metronomic cyclophosphamide (OMC) in ovarian cancer is effective both in combinations with targeted agents and in monotherapy, with an excellent toxicity profile [12, 13].

This retrospective observational study aims to evaluate the clinical benefit and objective response of OMC treatment in ovarian cancer patients, investigating potential predictive markers of response.

## Materials and methods

Ovarian cancer patients treated with metronomic cyclophosphamide between January 2017 and May 2022 at Academic Division Gynecology, Mauriziano Umberto I Hospital, Torino, Italy, were included in this retrospective observational study.

For each patient, the following data were collected from the hospital’s informatic archive and medical records: age, histology, FIGO stage, germline BRCA mutational status, surgical treatment, number of lines of previous therapy, PFI at first recurrence, performance status, and symptomatology reported by the physician.

The dose of oral cyclophosphamide prescribed was 50 mg daily continuously. 28 days of treatment was considered as one cycle, and any interruption or early discontinuation were reported. Chemotherapy-related toxicities were classified according to the Common Terminology Criteria for Adverse Events v. 5.0 (CTCAE).

Clinical examination and serum Ca125 measurement were performed on day 1 of each cycle. Radiological response was assessed through CT scan according to the RECIST 1.1 criteria every 3–6 months or earlier in case of clinical suspicion of progression or significant marker elevation. Biochemical response was defined applying the Gynecological Cancer Intergroup (GCIG) criteria. A reduction in Ca125 ≥ 50% from the baseline and maintained for at least 21 days was considered as biochemical response. Progression disease (PD) was defined as an increase in Ca125 ≥ 100% from the lowest value achieved during treatment. Stable disease (SD) was considered as a change in Ca125 that does not fit the disease progression or response criteria.

Radiological response according to RECIST criteria, reduction in Ca125, and improvement or absence of symptoms as reported by physicians were combined to define the clinical benefit of the treatment.

Patients were distributed into three subgroups: *responders* if there was a response to treatment, *non-responders* when this did not occur, and *long responders* if the response was maintained for ≥ 6 months. The variables age, performance status, and PFI at the first relapse were assessed in the three subgroups.

The study was conducted in accordance with the Declaration of Helsinki and approved by the Institutional Review Board and Ethics Committee A.O.U. Città della Salute e della Scienza di Torino/A.O. Ordine Mauriziano /ASL Città di Torino (registration number n° 0068594). In agreement with the Ethics Committee, it was not necessary to collect specific informed consent due to the retrospective nature of the study and the data analyzed.

### Statistical analysis

Data analysis was performed using SPSS Statistic 22.0 (IBM Inc., Chicago, IL, USA).

Response rate was evaluated in terms of clinical benefit by including partial responses, complete responses, and disease stabilization.

The overall survival (OS) was measured from the date of treatment initiation to the date of the patient’s death or last follow-up, using the Kaplan–Meier curve. The duration of response was calculated as the interval between the best response obtained during therapy and disease progression. The comparison analysis considered the following groups of patients: *responders, non-responders,* and *long responders*. The analyzed parameters were compared using Chi-square test and analysis of variance with ANOVA test and *p* < 0.05 was considered the cutoff for statistical significance.

## Results

A total of 38 ovarian cancer patients treated with metronomic cyclophosphamide in the first line or recurrent setting were included in the study. For 3/38 (7.9%) patients, the treatment was ongoing at the time of data collection.

## Population

Patients’ characteristics are described in Table 1.

**Table 1:** Characteristics of patients

	Total patients (*n* = 38)
Age at diagnosis	
Average (range)	67 (41–48)
Age at start of metronomic cyclophosphamide	
Average (range)	72 (49–88)
Histologic type *n* (%)	*n* = 38
Serous high grade	29 (76%)
Endometrioid	5 (13%)
Undifferentiated	2 (5.3%)
Mucinous	1 (2.6%)
Clear cells	1 (2.6%)
Stage *n* (%)	*n* = 38
I	2 (5.3%)
II	2 (5.3%)
III	22 (58%)
IV	12 (32%)
gBRCA status *n* (%)	*n* = 26 (68%)
gBRCA mut	2 (8%)
gBRCA wild type	24 (92%)
Surgery *n* (%)	*n* = 29 (76%)
PDS	17 (59%)
IDS	12 (41%)
Residual tumor macroscopically 0 (R0)	18 (62%)
Metronomic chemotherapy line *n* (%)	
I	2 (5.3%)
II	5 (13%)
III	7 (18%)
IV	8 (21%)
V	9 (24%)
VI	4 (11%)
VII	2 (5.3%)
VIII	1 (2.6%)
ECOG PS at the start of metronomic chemotherapy *n* (%)	
0	8 (21%)
1	22 (58%)
2	8 (21%)

*gBRCA* germinal *BRCA* *PDS* primary debulking surgery, *IDS* interval debulking surgery

Mean age at diagnosis was 67 years (range 41–88), and mean age at the beginning of metronomic chemotherapy was 72 years (range 49–88). BRCA mutational status was wild type in the majority (92%). Nine patients (24%) had no surgery as they were not considered suitable due to advanced age, performance status, and pluri-comorbidities.

The main histological type was high-grade serous epithelial carcinoma (76%) and FIGO stage at diagnosis was advanced (III/IV) in 34 patients (90%) while only 4 (10.6%) had early stage disease (I/II) at presentation.

The 68% of patients had PFI > 6 months at first relapse, while 31.4% had PFI ≤ 6 months. On average, three lines of treatment had been administered before metronomic therapy (range 0–7), in particular in 64% of cases, cyclophosphamide was administered after ≥ 3 previous lines of chemotherapy; in two patients (5.3%), metronomic cyclophosphamide was administered as the first-line treatment and in five patients (13%) as the second line, considering the patient’s age (≥ 80 years old), performance status, and comorbidities. At the beginning of the metronomic treatment, 21% of the patients had an ECOG PS 0, 58% ECOG PS 1, and 21% ECOG PS 2.

## Treatment response

The mean duration of treatment was 5.4 months (range 1–20). Thirty-five patients (92%) were evaluable for response having already done at least one radiological assessment or two clinical or biochemical evaluations. Response in terms of clinical benefit was obtained in 17 (48.6%) patients, 3 (8.6%) presented a partial response and 14 (40%) presented disease stability. No patient achieved a complete response. The median duration of response, considering partial response and disease stability, was 7.4 months (Table 2).

**Table 2:** Response to treatment

Clinical benefit	Evaluable patients	*n* = 35
	CR	0 (0%)
	PR	3 (8.6%)
	SD	14 (40%)
	PD	18 (51.4%)
Average duration of response (months)		7.4
Biochemical response (Ca125)	Evaluable patients	*n* = 35
After 1 month	PR	0 (0%)
	SD	25 (83.3%)
	PD	5 (16.7%)
After 3 months	PR	3 (10%)
	SD	13 (43.3%)
	PD	7 (23.3%)
After 6 months	PR	4 (13.3%)
	SD	3 (10%)
	PD	4 (13.3%)
Clinical response (absence/reduction of symptoms)	Evaluable patients	*n* = 35
After 1 month	YES	23 (66%)
	NO	12 (34%)
After 3 months	YES	18 (51%)
	NO	17 (49%)
After 6 months	YES	11 (31%)
	NO	24 (69%)
Radiological response		
After 3 months	Evaluable patients	*n* = 7
	PR/CR/SD	0 (0%)
	PD	7 (100%)
After 6 months	Evaluable patients	*n* = 12
	CR	0 (0%)
	PR	3 (25%)
	SD	3 (25%)
	PD	6 (50%)
Average duration of therapy (months)		5.4

*CR* complete response, *PR* partial response, *SD* stable disease, *PD* progression disease

Among the 30 patients whose response was assessed through Ca125, the mean value at the beginning of metronomic therapy was 1963 U/ml (range 12–45,283). Response based exclusively on Ca125 was obtained in 25/30 patients (83%) as stable disease after one cycle of treatment. After 3 treatment cycles, 3/30 patients (10%) had a partial response and 13/30 (43.3%) a disease stability, while after 6 treatment cycles, 4/30 (13.3%) reached a partial response and 3/30 (10%) a disease stability.

The clinical response in terms of absence or improvement of symptoms referred by the patient and reported by the physician was detected in 23/35 (66%) patients after 1 cycle of therapy, in 18/35 (51%) after 3 cycles, and in 11/35 (31%) after 6 cycles.

Nineteen women (54%) underwent radiological assessment at 3 or 6 months from the beginning of the treatment; all the seven patients evaluated at 3 months had disease progression according to the RECIST criteria, while for patient that underwent restaging at 6 months, 3/12 (25%) had a partial response, 3/12 (25%) had a stable disease, and 6/12 (50%) had progression disease.

### Toxicity

No G3-4 hematological toxicities were recorded. The main toxicities observed were asthenia G1–G2 in 13/38 (34%) and nausea in 5/38 (13%) patients; nephrological toxicity in terms of elevation of creatinine was recorded in 3/38 (8%) patients, and myalgias in 2/38 (5.3%) patients. Seven patients (18%) had at least one delay and only one woman (2.6%) interrupted treatment due to toxicity (nausea).

### Subgroup analysis and survival

Among the 35 patients evaluable for response, 18 (51.4%) were classifiable as *non-responders*, 9 (25.7%) as *responders*, and 8 (22.9%) as *long responders* (Table 3).

**Table 3:** Subgroup analysis: non-responders, responders, long responders

	Non responders	Responders	Long responders	*p* value
Patients *n* (%)	18 (51,4%)	9 (25.7%)	8 (22.9)	–
Age				
Average(range)	68 (45–86)	73 (55–86)	79 (68–88)	0.05
≤ 75 years n (%)	13 (65%)	5 (25%)	2 (10%)	0.08
≥ 75 years n (%)	5 (33.3%)	4 (26.7)	6 (40%)	
ECOG				
ECOG 0 n (%)	3 (42.9%)	3 (42.9%)	1 (14.3%)	0.54
ECOG 1 n (%)	12 (60%)	3 (15%)	5 (25%)	
ECOG 2 n (%)	3 (37.5%)	3 (37.5%)	2 (25%)	
Platinum sensitive at first recurrence n (%)	13 (54.2%)	6 (25%)	5 (20.8%)	0.87
Platinum resistant at first recurrence n (%)	5 (45.5%)	3 (27.3%)	3 (27.3%)	

Median age of *non-responders* was 68 years (range 45–86), for *responders*, it was 73 years (range 55–86), while the oldest patients were *long responders*, with a median age of 79 years (range 68–88; *p* = 0.05). In particular, the 66.7% of patients older than 75 years obtained a response to the treatment and in the 40% of cases, it was maintained for a period ≥ 6 months (*p* = 0.08).

Considering responses according to performance status, prolonged response for a period ≥ 6 months occurred in the 14.3% of patients with ECOG 0, the 25% of patients with ECOG 1, and 25% of patients with ECOG2 (*p* = 0.54).

Patients with PFI > 6 months after the first-line treatment were 25% responders and 20.8% long responders. The 54.6% of patients with PFI ≤ 6 months responded to metronomic treatment and the 27.3% were long responders (*p* = 0.87).

**Fig. 1:**
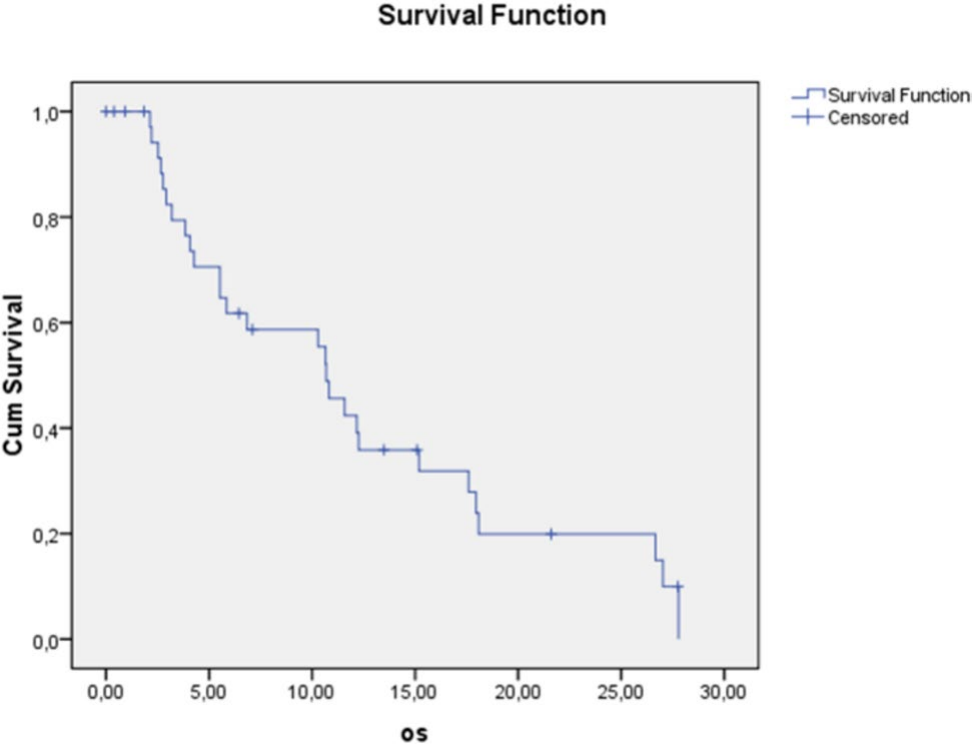
Overall survival

The median survival of the patients included was 10.6 months (CI 9.0–12.3), with no difference by age (Fig. 1).

## Discussion

In this retrospective study, the activity of OMC in ovarian cancer patients and its safety profile were confirmed. We can define the population investigated as elderly, highly pretreated, and with a non-optimal performance status: at the beginning of the metronomic treatment, the mean age was 72 years, 64% of the patients had undergone ≥ 3 lines of chemotherapy, and in 79% of the cases, the ECOG was ≥ 1.

The response to metronomic cyclophosphamide therapy was evaluated combining clinical, biochemical, and radiological criteria, with a particular attention to the clinical benefit in terms of symptomatic well-being. A clinical benefit to the treatment was obtained in 66% of patients after 1 month of therapy, in 51% after 3 months, and in 31% after 6 months.

In recent years, thanks to the introduction of new target drugs and maintenance strategies, patients affected by ovarian cancer often experience a chronicization and slow progression of their disease with a prolongation of life expectancy [14]. In our cohort, responses were more evident and durable for elderly patients with a poor performance status and regardless PFI at the first relapse.

In our population, the median survival was 10.6 months, similar to studies conducted on younger and less pretreated patients [15]. Previous studies questioned the clinical benefit of treatments after the fourth line; however, differences in terms of survival were found between the treated and untreated groups. Therefore, patient selection is crucial, and symptom control with a well-being assessment should be integrated into responses evaluation [5]. Questionnaires on the quality of life focused on the state of health, in particular the assessment of abdominal and gastrointestinal symptoms, have proven to be valid predictors of response to treatments in recurrent disease [7].

We obtained a response rate of 48.6% and a mean duration of the best response of 7.4 months. These data are comparable with those obtained by Ferrandina et al., evaluating highly pretreated patients undergoing OMC chemotherapy, demonstrating a clinical benefit in 40.7% and a duration of response ≥ 6 months [16]. Spiliopoulou et al. obtained an objective response rate of 48% and a progression-free survival of 2.6 months, but with significant differences between platinum-sensitive and platinum-resistant patients, 8.2 and 2.4 months, respectively [15]. In the most recently published real-life experience, the disease control rate reported was 47%, higher in low-grade indolent tumors, and the progression-free survival was 3.5 month [17]. Even a review conducted through March 2023 showed an objective response rate (ORR) and disease control rate for recurrent or platinum-refractory ovarian cancer of 18.8% and 36.2%, respectively, and OS of 8.7 months [18]. All these studies were retrospective and with limited samples; however, they reflected the local realities; our investigation is characterized by the oldest and most pretreated population.

R*esponders, non-responders*, and *long responders* characteristics were evaluated, to identify any predictive factors of response.

Women over 75 years old responded in 66.7% of cases and *long responder* patients were the oldest, with a mean age of 79 years (*p* = 0.05). This data could relate to the fact that all the women undergoing metronomic chemotherapy as I or II line of treatment were aged ≥ 80 years. Elderly patients are underrepresented in clinical trials and, consequently, data on the feasibility and tolerability of chemotherapy are often inadequate [19, 20]. Using standard chemotherapy regimens could expose them to dose reductions and interruptions, with a negative impact on survival [21]; therefore, a frailty assessment is crucial, to prevent treatment-related toxicities and, at the same time, undertreatment [22]. Consistent with previous studies [15, 16, 23], in our experience, OMC demonstrated an excellent toxicity profile, with no significant hematological toxicities and only one patient underwent treatment discontinuation due to nausea. Therefore, OMC can be a valid option also in the elderly population.

In our cohort, performance status did not significantly correlate with the response to treatment, but 25% of ECOG 1 patients and 25% of ECOG 2 were long responders (*p* = 0.54): we can consider this treatment regimen active even in the most compromised patients. In a 2013 Asian study, ECOG seemed to correlate with patient quality of life; in the setting of refractory or recurrent epithelial ovarian cancer, patients receiving chemotherapy had quality of life questionnaire scores comparable to patients referred to palliative care [24]. However, to consider the patients’ wishes is crucial, as most of them prefer to undergo subsequent treatments even if a minimal survival benefit is expected [25].

In our experience, 54.6% of platinum non-eligible patients after the first line of therapy responded to metronomic treatment, in particular 27.3% maintained the response for more than 6 months (*p* = 0.87); moreover, the majority of our population were germline BRCA wild type. The alkylating effect of cyclophosphamide could explain its activity also in this category of patients, overcoming resistance to platinum and becoming an effective treatment alternative [26].

The retrospective nature of the study did not allow the administration of questionnaires on quality of life. However, the low rate of radiological examinations performed reflected a close attention to patients’ symptoms in real-life clinical practice; for highly pretreated and elderly patients, instrumental restaging was subordinated to Ca125 values and to the symptoms reported.

In light of these results, it seems appropriate to prefer metronomic cyclophosphamide in the treatment of women with limited therapeutic alternatives, especially if elderly. Prospective clinical trials should investigate its activity as an early therapeutic option.

Metronomic chemotherapy has an anti-angiogenic mechanism of action which is mainly expressed through a direct action on endothelial cells [27]. OMC has also demonstrated an immunological effect, inducing reduction of circulating Treg lymphocytes and restoring natural killer cell activity, with immunological optimization of the tumor microenvironment [11]. The combined action of cyclophosphamide with anti-angiogenics such as bevacizumab is known, and the benefit of maintenance therapy after standard chemotherapy has been demonstrated [28]. Considering the effect in boosting the immune response against tumors [29], future studies should consider the association of OMC to immunotherapy.

## Conclusion

This study demonstrated the clinical benefit of oral metronomic cyclophosphamide treatment in recurrent ovarian cancer and its feasibility in clinical practice, due to the good toxicity profile. Furthermore, our experience highlighted the importance of assessing the patient's well-being with an emphasis on quality of life. Therefore, prospective studies integrating specific patients-reported outcomes are needed, to optimize the clinical benefit assessment, especially in elderly and treatment settings in which the therapeutic alternatives can give limited benefits.

## Data Availability

The datasets generated during the current study are available from the corresponding author on reasonable request.
